# Mixed T-cell chimerism at 3 months predicts late relapse in acute myeloid leukemia after allogeneic stem cell transplantation

**DOI:** 10.1038/s41598-026-69047-8

**Published:** 2026-09-04

**Authors:** Mikael Lisak, Malin Nicklasson, Robert Palmason, Stina Wichert, Anders Eivind Myhre, Trym Døviken, Cecila Isaksson, Kristina Carlson, Johan Törlén, Per-Ola Andersson, Jan-Erik Johansson, Markus Hansson

**Affiliations:** 1 Department of Hematology and Coagulation, Sahlgrenska University Hospital, Gothenburg, Sweden; 2 Sahlgrenska Academy at Gothenburg University, Gothenburg, Sweden; 3 Department of Hematology, Skane University Hospital, Lund, Sweden; 4 Department of Hematology, Oslo University Hospital, Oslo, Norway; 5 Department of Hematology, Norrland University Hospital, Umeå, Sweden; 6 Department of Hematology, Uppsala University Hospital, Uppsala, Sweden; 7 Department of Cell Therapy and Allogeneic Stem Cell Transplantation, Karolinska University Hospital, Stockholm, Sweden

**Keywords:** Acute myeloid leukemia, Allogeneic stem cell transplantation, T-cell chimerism, Measurable residual disease, Relapse, Immune reconstitution, Cancer, Immunology, Oncology

## Abstract

Relapse remains the leading cause of treatment failure after allogeneic stem cell transplantation (HCT) in acute myeloid leukemia (AML), yet predictors of late relapse remain poorly defined. We conducted a multicenter retrospective study including 388 patients with AML, representing a disease-specific cohort undergoing first HCT across six Scandinavian centers to evaluate the prognostic role of early T-cell chimerism assessed at three months post-HCT. Mixed T-cell chimerism (MTC) was defined as < 95% donor-derived CD3 + T cells. Among patients alive and relapse-free at 12 months, three-month MTC was associated with a significantly higher 5-year cumulative incidence of relapse compared with complete T-cell chimerism (29% vs 17%, p = 0.024) and inferior 5-year RFS (62% vs 74%, p = 0.017). In contrast, pre-HCT measurable residual disease (MRD) was primarily associated with early relapse and showed a weaker association with relapse beyond 12 months. Exploratory analyses suggested that the adverse impact of pre-HCT MRD-positivity was most pronounced among patients with MTC. These findings indicate a temporal distinction in relapse dynamics, in which residual disease burden drives early relapse, whereas impaired immune reconstitution is associated with late relapse. Early T-cell chimerism may therefore identify patients at increased risk of late relapse and could contribute to improved post-HCT risk stratification.

**Supplementary Information:**

The online version contains supplementary material available at https://doi.org/10.1038/s41598-026-69047-8.

## Introduction

The curative potential of allogeneic stem cell transplantation (HCT) in acute myeloid leukemia (AML) relies on both the cytotoxic effect of conditioning and the immunological graft-versus-leukemia (GvL) effect, primarily mediated by donor-derived T cells^1^. Despite this, relapse remains the leading cause of treatment failure and death after HCT^2–4^. Established risk factors for relapse include genetic risk, disease stage at transplant and measurable residual disease (MRD)^5–8^.

Considerable emphasis has been placed on identifying sensitive tools for detection of incipient signs of AML to enable early interventions against relapse. Regardless of method, pre-^9,10^ and post-HCT MRD^7,8^ has been associated with increased risk of relapse. However, its applicability is limited by the absence of specific markers in a subset of patients and by clonal evolution over time^11,12^. In such cases, changes in donor chimerism may provide additional information on disease dynamics.

Beyond disease burden, relapse risk after HCT is influenced by the strength of the GvL effect^13,14^. T-cell chimerism reflects the balance between donor and recipient immunity and has been proposed as a surrogate marker of immune-mediated disease control. Mixed T-cell chimerism (MTC), even at early time points such as 1–3 months post-HCT, may indicate impaired immune reconstitution and reduced GvL activity^15–17^. However, studies evaluating the prognostic impact of chimerism have shown heterogeneous results, likely due to differences in patient populations, timing and analytical methods^18–21^.

Recent work suggests that combining MRD and T-cell chimerism may improve post-HCT risk stratification^8,15,22^. Nevertheless, most studies have focused on relapse risk in general or on early post-HCT outcomes. In contrast, late relapse occurring beyond the first year after HCT remains less well characterized, despite representing a clinically significant proportion of events^23^.

Full reconstitution of T cells after HCT can take months to years, especially when taking both quantity and function into consideration^14,24^.

Previous studies demonstrate that early relapses are more common amongst patients with pre-HCT MRD-positivity highlighting its importance for short-term risk prediction^25^.

We hypothesized that early T-cell chimerism reflects long-term immune control and is associated with late relapse risk. The aim of this study was therefore to investigate whether T-cell chimerism at three months post-HCT is associated with late relapse (beyond 12 months after HCT), and to explore its relationship with pre-HCT MRD in patients receiving reduced intensity or myeloablative conditioning regimens (RIC, MAC).

## Methods

### Patients

Patients underwent HCT for AML between 2010 and 2016 at five transplant centers in Sweden (Sahlgrenska University Hospital, Gothenburg; Skåne University Hospital, Lund; Karolinska University Hospital, Stockholm; Uppsala University Hospital, Uppsala and Norrland University Hospital, Umeå) and one in Norway (Oslo University Hospital, Oslo).

Eligible patients were ≥ 15 years with AML undergoing HCT between 2010 and 2016. The exclusion criteria were: 1) haploidentical donor, 2) cord blood cell transplant, 3) previous HCT, 4 ) no T-cell chimerism status at three months, or 5) not in complete morphological remission (CR) at three months, post-HCT.

The study was approved by the Gothenburg Ethical Review Authority (“Regionala etikprövningsnämnden i Göteborg”, Dnr 144-18), and for this retrospective study, informed consent was waived. All research was performed in accordance with relevant guidelines/regulations and in accordance with the Declaration of Helsinki.

### Data collection

Clinical data were collected from medical records and local transplant registries at each participating HCT-site.

### T-cell chimerism

T-cell chimerism was assessed in peripheral blood (PB) and/or bone marrow (BM) at the routine three-month evaluation. The time-point corresponded to routine clinical follow-up, including parallel evaluation of remission status, and when available, MRD assessment.

Lineage-specific cell separation was performed, and donor chimerism was analyzed using short tandem repeat (STR)-based methods and, in some cases, quantitative PCR (qPCR). Chimerism was expressed as percentage of donor-derived DNA. Additional methodological details are provided in the Supplementary Methods.

Patients were classified according to their three-month post-HCT T-cell chimerism status. Complete T-cell chimerism was defined as  95% donor CD3 + T cells at the routine three-month assessment, consistent with prior studies^8,15,22,26^ in AML/myelodysplastic syndrome (MDS), and MTC as < 95% donor T cells in either blood or bone marrow. Throughout the manuscript, the terms complete T-cell chimerism (CTC) and MTC refer to T-cell chimerism assessed at this three-month time point unless otherwise specified.

### AML relapse definition and risk categorization

AML relapse was defined as the reappearance of leukemia after achieving remission, based on bone marrow blasts ≥ 5%, circulating blasts on ≥ 2 samples, or extramedullary disease.

Genetic risk at diagnosis was classified according to the European LeukemiaNet (ELN) 2017^27^ risk criteria. Relapse risk at transplantation was categorized as low (LR), intermediate (IR) or high (HR), according to Swedish National Guidelines^28^ (Supplementary Methods).

When flow cytometry (FCM) was used to assess MRD status, the cut-off ≥ 0.1% was regarded as MRD-positive. When PCR was used simultaneously with FCM, both had to be negative to classify as MRD-negative.

### Graft-versus-host disease

Acute GvHD (aGvHD) was based on medical records and defined and graded according to the modified Glucksberg criteria^29^. The National Institute of Health (NIH) Consensus Development Project 2014 guidelines were used to define and score chronic GvHD (cGvHD) in each involved organ^30^, and was registered within the specified time period or until relapse or death.

GvHD prophylaxis was administrated according to local protocols (see Supplementary Methods).

### Conditioning intensity

Conditioning intensity (RIC or MAC) was defined according to Bacigalupo et al.^31^. See Supplementary Methods for details in conditioning regimens and classification of treosulfan-based conditioning.

### Study endpoints

The primary endpoint was the cumulative incidence of relapse (CIR) at 60 months from landmark, including only patients in CR at the landmark. The primary landmark was at 12 months post-HCT (L12), and the secondary landmark at 3 months (L3).

Secondary endpoints included aGvHD, cGvHD, relapse-free survival (RFS), non-relapse mortality (NRM) and overall survival (OS). Relapse-free survival was defined as time to relapse or death, and NRM as death without prior relapse.

### Statistical analysis

Chi-square (or Fisher´s exact test) and Mann–Whitney U-test were used for ordinal data or if skewed distribution to summarize baseline characteristics and outcome. The median follow-up time was estimated by the reverse Kaplan–Meier method^32,33^. Time-to-event outcomes were analyzed using survival methods and within a pre-specified landmark framework at 3 (L3) or 12 (L12) months post-HCT. Only patients alive and event-free at each landmark were included. Cumulative incidence of relapse was analyzed using Fine-Gray subdistribution hazard models with NRM as a competing event. Cumulative incidence function curves were generated using Stata software (see below), and group differences were evaluated using Gray’s test^34,35^. Relapse-free survival and OS were analyzed using Cox proportional hazards models within the same landmark framework. Patients were censored at last follow-up or at 60 months after the landmark, whichever occurred first. Kaplan–Meier curves were used to estimate RFS and OS. Group comparisons were performed using the log-rank test.

For the analyses of CIR, RFS and OS, the following variables were evaluated in the univariable regression analyses: T-cell chimerism status (MTC vs CTC), pre-HCT MRD status (positive vs negative), disease risk at HCT (low/intermediate vs high), in vivo T-cell depletion (yes vs no), conditioning intensity (RIC vs MAC), cGvHD (yes vs no), HLA-matching (8/8 vs < 8/8), stem cell source (PBSC vs BM), recipient CMV serostatus (positive vs negative), and patient age at HCT.

Multivariable models included T-cell chimerism together with the following prespecified clinically relevant covariates: pre-HCT MRD status, disease risk at HCT, cGvHD, conditioning intensity, and T-cell depletion.

**Fig. 1:**
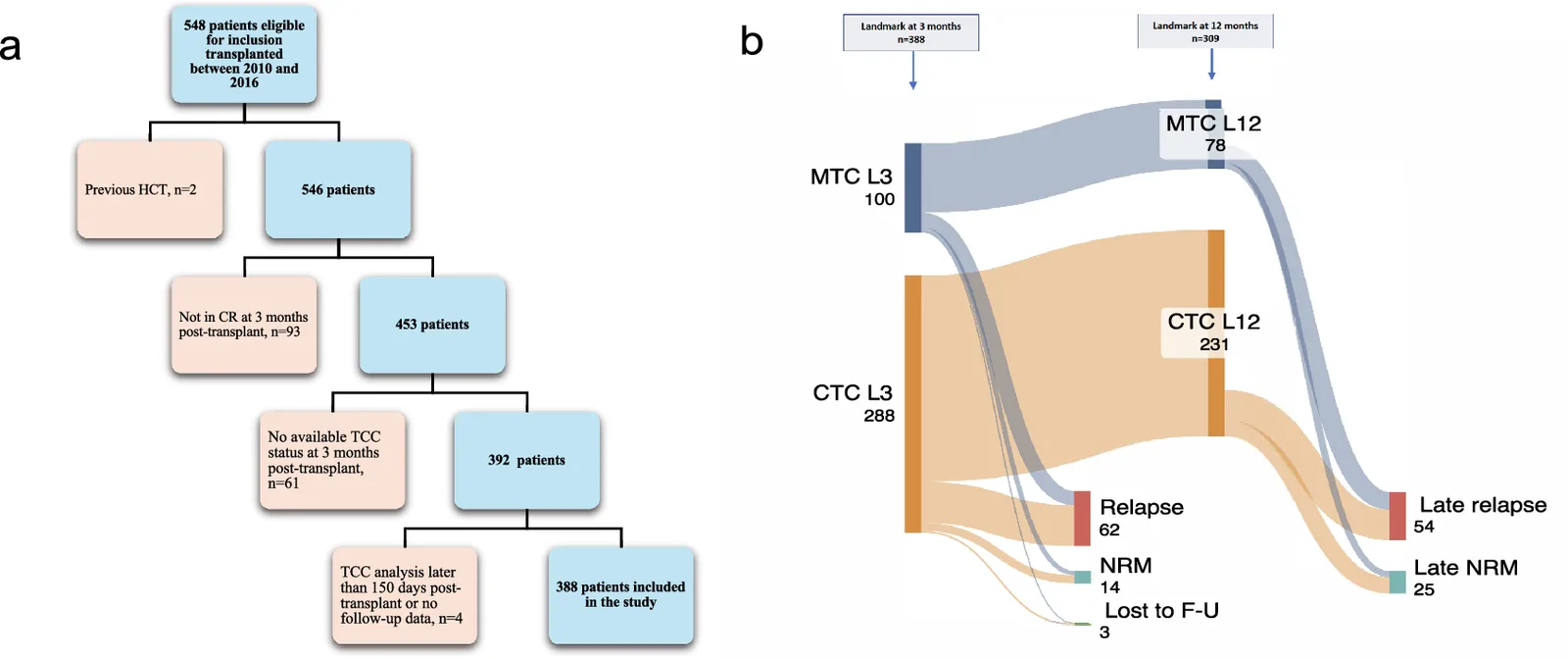
Study cohort and landmark populations. Flow diagram illustrating (**a**) screening, inclusion and exclusion and (**b**) patient flow from the 3-month landmark (L3) to the 12-month landmark (L12) and subsequent outcomes. CTC – complete T-cell chimerism; F-U – follow-up HCT – allogeneic hematopoietic stem cell transplantation; L3 – 3-month landmark; L12 – 12-month landmark; MTC – mixed T-cell chimerism; NRM – non-relapse mortality.

In the model building, each predictor was first evaluated in univariable models. Multivariable models included clinically relevant covariates selected a priori together with additional variables selected on the basis of univarialbe analyses, to assess independence of effects and potential confounding. Confounding was evaluated using a change-in-estimate approach. A change in HR or SHR > 10% after adjustment was considered indicative of confounding. Interaction (effect modification) was evaluated using a likelihood-ratio test comparing regression models with and without the interaction term. Proportional hazards assumptions were assessed using Schoenfeld residuals. All tests and confidence intervals were two-sided, and p-values less than 0.05 were considered statistically significant. For comparison of background characteristics, the SPSS version 30 (IBM® SPSS® Statistics, NY, USA) was used. The survival and cumulative incidence analyses were performed using Stata (StataCorp, College Station, TX) version 19.5. Figure 1B was generated using SankeyMATIC (https://sankeymatic.com).

Additional details regarding statistical methods are provided in the Supplementary Methods.

## Results

A total of 388 patients were included in the study. Of these, 309 were alive and in CR at 12 months post-HCT and comprised the primary landmark cohort. Among patients who were excluded between L3 and L12, the distribution of MTC and CTC did not differ from the overall cohort (p = 0.70). Among the 62 patients who relapsed between L3 and L12, 16 (26%) had MTC and 46 (74%) CTC, mirroring the distribution in the overall L3 cohort, in which 100 of 388 patients (26) had MTC and 288 (74%) had CTC. See Fig. 1a,b.

The median age at HCT was 56 (range: 15—71) years. Patients in the CTC group were younger than those in the 3-month MTC group (median 54 versus 59 years, p = 0.036), although the difference was not significant among patients in CR at 12 months. No significant differences were observed in pre- or post-HCT MRD status between groups. Testing of MRD at three months was less frequent in patients with MTC (44% vs 33%, p = 0.041), but this difference was not observed among patients in CR at 12.

T-cell depletion and MAC was more common in the CTC group (77% vs 62%, p = 0.033 and 49% vs 35%, p = 0.0015), whereas MTC was more common in patients receiving RIC (33% vs 19%). HLA-mismatched donors were more common in the CTC group (p = 0.0014). T-cell chimerism was assessed in PB (43%), BM (7%), or both (50%), with some discordance observed between compartments (see Supplementary Results).

The median follow-up time was 60 months (95% CI, 52–70). See Tables 1 and 2 for patient characteristics and results.

**Table 1:** Background characteristics (n = 388).

Characteristic	Mixed chimerism n = 100	Full chimerism n = 288	P value
Age at HCT, median (range), yrs	59 (17 – 71)	54 (15 – 71)	*p* = *0.036*
Females – n (%)	50 (50)	132 (46)	*p* = *0.52*
HCT-CI score – n (%)			
0–2	56 (56)	142 (49)	*p* = *0.63*
3–5	24 (24)	70 (24)	
≥ 6	2 (2)	10 (3)	
Unknown	18 (18)	66 (23)	*p* = *0.30*
Disease risk group – n (%)			
High	71 (71)*	202 (70)	*p* = *0.77*
Cytogenetic/Mutation High	26 (26)^#^	86 (30)^§^	*p* = *0.77*
Secondary AML	16 (16)	67 (23)*	*p* = *0.12*
Disease stage at HCT – n (%)			
CR1	74 (74)	229 (80)*	*p* = *0.25*
CR2	17 (17)	38 (14)*	*p* = *0.35*
> CR2	1 (1)	1 (1)*	*n.s*
Non-CR	6 (6)	15 (6)*	*p* = *0.77*
MRD at HCT (among patients in CR at HCT) – n (%)			
Positive (of available MRD)	14/50 (18)	34/135 (25)	*p* = *0.70*
Unknown (of patients in CR)	44/94 (47)	137/272 (50)	*p* = *0.55*
MRD at 3 months post-HCT (among patients in CR at 3 months) – n (%)			
Positive (of available MRD)	7/56 (13)	15/194 (8)	*p* = *0.27*
Unknown (of patients in CR)	44/100 (44)	94/288 (33)	*p* = *0.041*
Chimerism method – n (%)			
STR + PCR	18 + 61 = 79 (79)	11 + 162 = 173 (60)	*p* = *0.86*
FISH (RD)	2 (2)	5 (2)	
Unknown	19 (19)	110 (38)	*p* < *0.001*
Stem cell source – n (%)			
BM	11 (11)	17 (6)	*p* = *0.090*
PBSC	89 (89)	271 (94)	
Donor type – n (%)			
RD	20 (20)	59 (20)	*p* = *0.92*
URD	80 (80)	229 (80)	
HLA matching – n (%)			
10/10	93 (93)	245 (85)*	*p* = *0.048*
≤ 9/10	7 (7)	42 (15)	
≤ 7/8	3 (3)	31 (11)^κ^	*p* = *0.014*
Matching (patient/donor) – n (%)			
Male/Female	8 (8)	35 (12)	*p* = *0.25*
All other combinations	92 (92)	252 (88)*	
CMV IgG-pos recipients –n (%)	69 (69)*	223 (77)^#^	*p* = *0.10*
Donor age, median (range), years	29 (19 – 71)	29 (18 – 67)^Φ^	*p* = *0.83*
Conditioning – n (%)			
RIC	65 (65)	134 (51)	*p* = *0.0015*
MAC	35 (35)	154 (49)	
T-cell depletion – n (% of ATG)			
ATG-Fresenius®/Grafalon®	4 (4)	23 (8)	*p* = *0.18*
30 mg/kg	0 (0)	1 (4)	*n.s*
40 mg/kg	4 (100)	22 (96)	
Thymo®	58 (58)	197 (68)	*p* = *0.059*
≤ 5 mg/kg	39 (67)	108 (55)	*p* = *0.092*
> 5 mg/kg	19 (33)	89 (45)	
Campath	0 (0)	2 (1)	*n.s*
No ATG/T-cell depletion	38 (38)	66 (23)	p = 0.0033

ATG – anti-thymocyte globulinacute; BM – bone marrow; CMV – cytomegalovirus; CR – complete remission; CR1, CR2 or > CR2 – first or succeeding complete remission; eGFR – estimated glomerular filtratrion rate; HCT-CI – hematopoietic cell transplantation comorbidity index; HCT – allogeneic stem cell transplantation; MAC – myeloablative conditioning; MRD – measurable residual disease; PBSC – peripheral blood stem cells; RD – related donor; RIC – reduced intensity conditioning; TBI – total body irradiation; Treo – treosulfan; Yrs – years; URD – unrelated donor.

*Data missing for 1 patient.

^#^Data missing for 2 patients.

^κ^Data missing for 3 patients.

^Φ^Data missing for 6 patients.

^§^Data missing for 13 patients.

**Table 2:** Results (n = 388).

Variable	Mixed chimerism n = 100	Full chimerism n = 288	P value
Acute GvHD – n (%)			
Any Grade	49 (49)	174 (60)	*p* = *0.13*
Grade I	24 (49)	85 (49)	*p* = *0.31*
Grade II	19 (39)	72 (41)	
Grade III	4 (8)	16 (9)	
Grade IV	2 (4)	1 (1)	
None – Grade I	75 (75)	199 (69)	*p* = *0.26*
Grade II –Grade IV	25 (25)	89 (31)	
None – Grade II	94 (94)	271 (94)	*p* = *0.97*
Grade III – Grade IV	6 (6)	17 (6)	
Chronic GvHD – n (%)			
Any Grade, within 12 mos after HCT	62 (62)^\xFE^	159 (55)*^@^	*p* = *0.24*
Moderate – severe	43 (43)	100 (35)^§@^	*p* = *0.23*
Infections after HCT – n (%)			
CMV treatment within first year	30 (30)*	139 (48)	*p* = *0.0015*
EBV treatment within first year	7 (7)*	25 (9)^Φ^	*p* = *0.60*
Invasive fungal infection	10 (10)	21(7)	*p* = *0.52*
DLI – n (%)	29 (29)*	29 (10)*	*p* < *0.001*
Relapse – n (%)	38 (38)	88 (31)	*p* = *0.17*
Late relapse (12–72 mos post-HCT) – n (%)			
Among patients in CR until 12 mos	20/78 (26)	34/231 (15)	*p* = *0.028*
Death – n (%)	43 (43)	103 (365)	*p* = *0.20*
Late death (12–72 mos post-HCT) – n (%)			
Among patients in CR until 12 mos	21/78 (27)	41/231 (18)	*p* = *0.080*
Cause of death – n (% of deaths)			
Relapse	28 (65)	69 (67)	*p* = *0.83*
GvHD or related infections	6 (14)	9 (9)	*p* = *0.34*
Infection	7 (16)	8 (8)	*p* = *0.12*
Other/unknown	2 (5)	17 (17)	*p* = *0.061*
Cause of late death (12–72 mos post-HCT) – n (% of late deaths)			
Relapse	14 (67)	23 (56)	*p* = *0.42*
GvHD or related infections	2 (10)	6 (15)	*p* = *0.71*
Infection	4 (19)	3 (7)	*p* = *0.17*
Other/unknown	1 (5)	9 (22)	*p* = *0.084*

CMV – cytomegalovirus; CR – complete remission; DLI – donor lymphocyte infusion; EBV – Epstein-Barr virus; GVHD – graft-versus-host disease; HCT – allogeneic stem cell transplantation.

*Data missing for 1 patient.

^§^Data missing for 4 patients.

^Φ^Data missing for 6 patients.

^\xFE^2 developed GvHD post DLI, administrated due to chimerism.

^@^4 developed GvHD post DLI, administrated due to chimerism.

### Relapse

Overall, 32% of the patients relapsed, of which half occurred beyond 12 months post-HCT.

#### T-cell chimerism status and late relapse

Among all patients, relapse occurred in 38% in the three-month MTC group and 31% in the CTC group (p = 0.17). Among the 309 patients that were alive and in CR at 12 months, 26% in the MTC group compared with 15% in the CTC group (p = 0.028; Table 2).

**Fig. 2:**
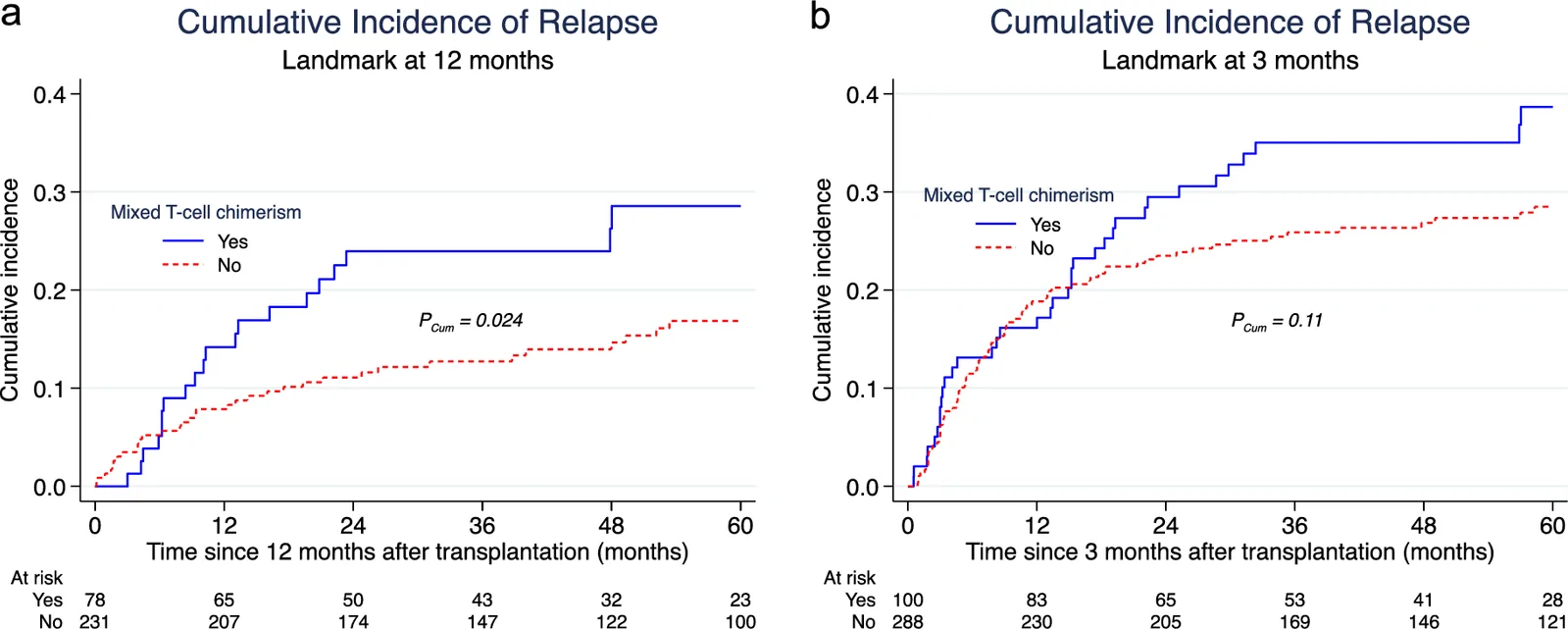
Cumulative incidence of relapse by T-cell chimerism status. Shown from (**a**) 12-month and (**b**) 3-month landmarks. Death was treated as a competing event.

The 60-month CIR from L12 was 29% (95% CI, 19–41) in the MTC group and 17% (95% CI, 12–23) in the CTC group (p = 0.024), whereas no significant difference was observed from L3 (39% [95% CI, 29–50] versus 28% [95% CI, 23–34], p = 0.11; Fig. 2A,B).

#### Pre-transplant MRD-status and early relapse

**Fig. 3:**
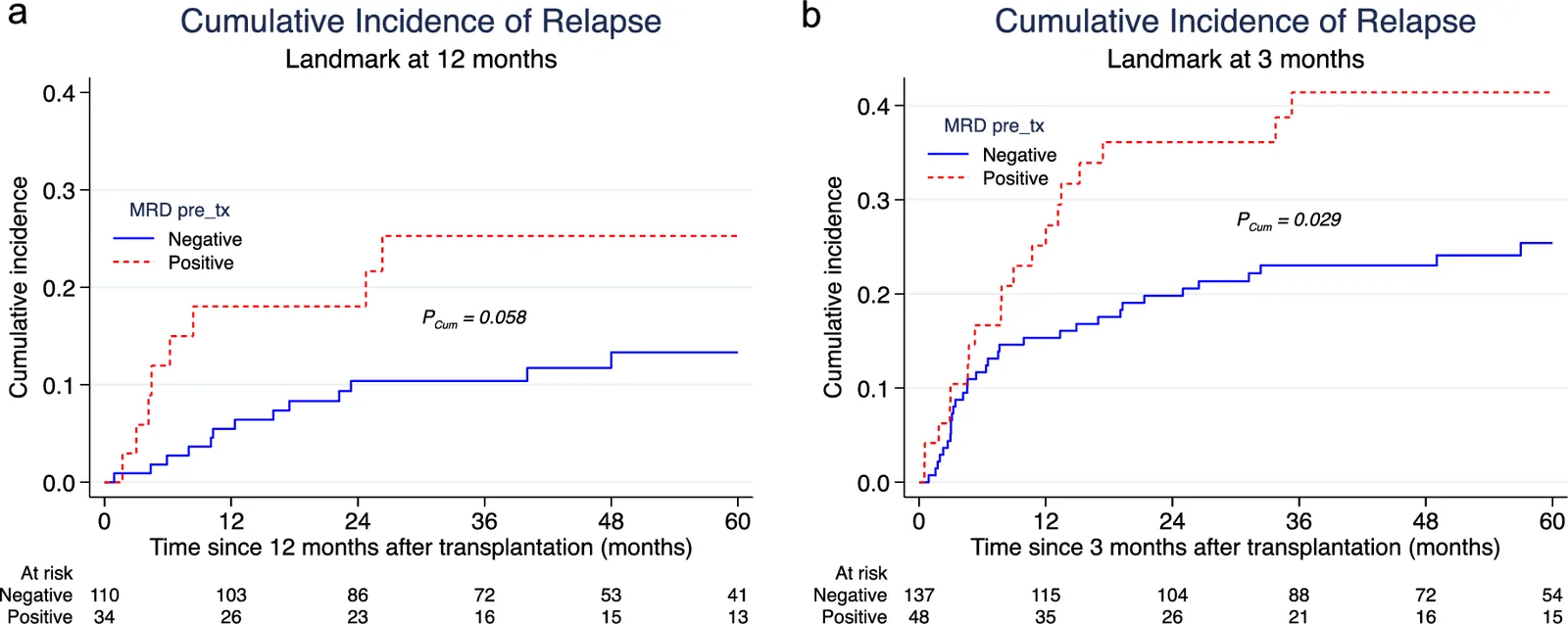
Cumulative incidence of relapse by pre-HCT MRD status. Shown from (**a**) 12-month and (**b**) 3-month landmarks. Death was treated as a competing event.

Pre-transplant MRD-positivity was associated with increased CIR from L3 (41% [95% CI, 29–57] versus 25% [95% CI, 19–34], p = 0.029), whereas the association from L12 was weaker and not statistically significant (25% [95% CI, 13–44] versus 13% [95% CI, 8–22], p = 0.058); Fig. 3A,B).

In univariable competing-risk regression, T-cell chimerism was the only variable significantly associated with CIR after L12, with lower relapse risk observed in the CTC group (subhazard ratio [SHR], 0.54, 95% CI, 0.31–0.93, p = 0.028). Pre-transplant MRD-positivity (SHR 2.28, p = 0.067), high risk disease at HCT (SHR 1.64, p = 0.13) and absence of cGvHD (SHR 1.57, p = 0.095) showed a numerically increased, but non-significant, relapse risk after L12. No associations were observed for conditioning intensity, T-cell depletion, donor match, stem cell source, CMV status or patient age at HCT.

In multivariable analyses adjusting for pre-HCT MRD status, disease risk at HCT, cGvHD, conditioning intensity and T-cell depletion, the association between T-cell chimerism and CIR remained. No significant interactions were observed between T-cell chimerism and pre-HCT MRD (p = 0.44) or other variables.

### RFS, NRM and overall survival

**Fig. 4:**
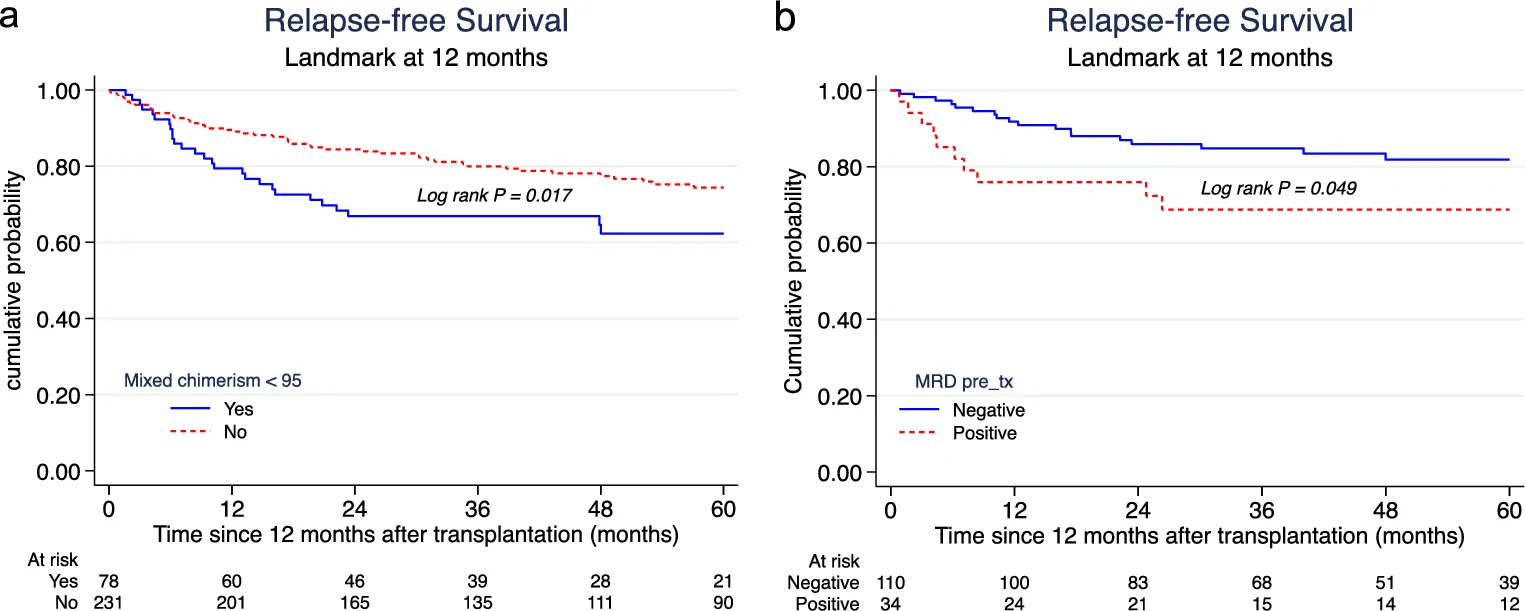
Relapse-free survival from the 12-month landmark. Stratified by (**a**) T-cell chimerism status and (**b**) MRD status.

The 60-months RFS from L12 was lower in patients classified as having MTC at three months compared with CTC (62% [95% CI, 49 –73] versus 74% [95% CI, 67 –80], p = 0.017). Similarly, pre-HCT MRD-positive compared with MRD-negative patients had inferior RFS (68% [95% CI, 49–82] versus 82% [95% CI, 72–88], p = 0.049; Fig. 4A,B).

In univariable analysis, T-cell chimerism was the only variable significantly associated with RFS after L12; CTC versus MTC group (hazard ratio [HR], 0.57, 95% CI, 0.36–0.91, p = 0.019). Pre-transplant MRD-positivity (HR 2.13, p = 0.055) and disease risk at HCT (HR 1.57, p = 0.083) showed non-significant trends towards inferior RFS. No significant associations were observed for other clinical variables included in the CIR analysis, and no significant interactions were identified.

No differences in NRM were seen between the MTC and the CTC group at 60 months from either L12 or L3 (Figure S2A,B).

**Fig. 5:**
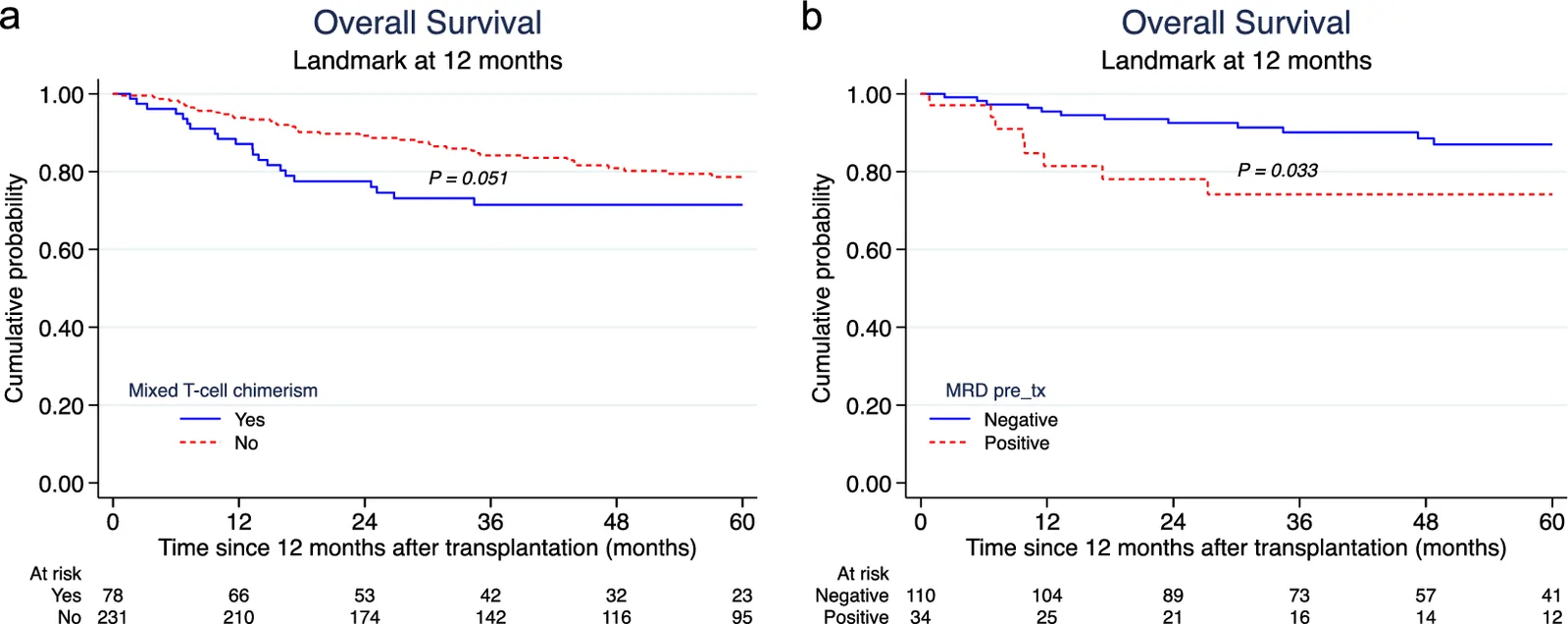
Overall survival by T-cell chimerism status. Shown from (**a**) 12-month and (**b**) 3-month landmarks.

Among patients in CR at 12 months, OS was lower in the MTC group compared with the CTC group (71% [95% CI, 60–80] versus 79% [95% CI, 72–84], p = 0.051). Pre-transplant MRD-positive patients had inferior OS compared with MRD-negative patients (74% [95% CI, 55–86] vs 87% [95% CI, 78–93], p = 0.033; Fig. 5A,B).

In the overall survival analysis, a significant interaction was observed between T-cell chimerism and pre-HCT MRD status. Pre-transplant MRD-positivity was associated with an increased risk of death among patients with MTC (HR 8.40, 95% CI 1.99–35.55), whereas no clear effect was observed among patients with CTC (HR 1.31, 95% CI 0.27–6.52).

**Fig. 6:**
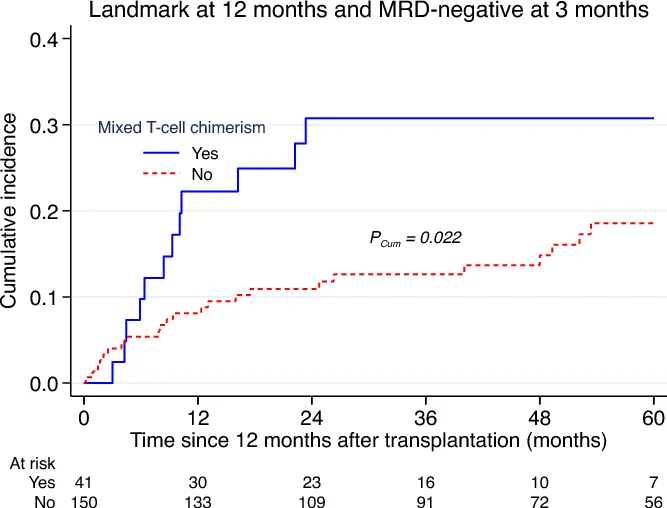
Cumulative incidence of relapse in MRD-negative patients at 3 months post-HCT, stratified by by T-cell chimerism status.

Subanalyses were made of patients in MRD-negative CR at three months post-HCT, to exclude that the T-cell chimerism status analyzed simultaneously, was affected by residual disease. The difference in CIR between MTC and CTC groups remained (Fig. 6) among MRD-negative patients.

### Time to relapse and from relapse to death

Patients with pre-HCT MRD-positivity showed numerically shorter time to relapse, and from relapse to death, compared with patients with three-month MTC, although differences were not statistically significant (Figure S3).

### Donor lymphocyte infusions

Donor lymphocyte infusions (DLI) were more frequent among patients with MTC compared with CTC (29% vs 10%; p < 0.001).

### Infections

Treatment of CMV reactivation was more common in the CTC group (48% vs 30%, p = 0.0015). No differences were observed in EBV or fungal infections.

### Acute and chronic GvHD

No significant differences were observed between the groups regarding the rate or severity of aGvHD; grade II–IV (25% vs 31%; p = 0.26) and grade III–IV (6% vs 6%; p = 0.97). No significant differences in rates or severity of cGvHD was seen between the MTC and CTC groups.

## Discussion

In this study, mixed T-cell chimerism (MTC) at three months post-HCT was associated with a higher cumulative incidence of late AML relapse beyond 12 months and inferior relapse-free survival compared with complete chimerism. In contrast, the adverse prognostic impact of pre-HCT measurable residual disease (MRD) was most pronounced in the early post-HCT period. These findings indicate a temporal distinction in which early relapse is primarily driven by residual disease burden, whereas late relapse is more strongly associated with impaired immune reconstitution.

Our data indicate that the prognostic impact of T-cell chimerism emerges predominantly in the late post-HCT phase, shifting the focus from overall relapse risk to the specific identification of patients at risk of late relapse. Biologically, MTC may reflect impaired immune reconstitution, increased tolerance, or reduced alloreactivity, allowing slowly expanding malignant or pre-leukemic clones to evade immune control and eventually manifest as late relapse. This interpretation is consistent with the established role of graft-versus-leukemia (GvL) immunity^1,13^ and delayed T-cell reconstitution following transplantation^24^. Early dominance of donor-derived T cells may therefore be critical for maintaining effective long-term surveillance.

In contrast, pre-HCT MRD reflects leukemic burden at the time of transplantation and is therefore most strongly associated with early relapse^5,36^. Although MRD-positive patients also showed a trend toward increased late relapse in this study, its dominant prognostic effect manifested early and persisted over time. The primary findings regarding MTC were independent of MRD availability, and exploratory analyses further suggested a meaningful interaction between MRD and chimerism status. Notably, the adverse prognostic impact of pre-HCT MRD on overall survival appeared most pronounced among patients with MTC, whereas complete T-cell chimerism (CTC) attenuated this risk. These findings are in line with previous studies demonstrating that combined assessment of MRD and T-cell chimerism improves post-HCT risk stratification^8^ and further support the concept of late relapse as a distinct clinical entity^23^.

Although conventional STR-based chimerism analysis was primarily used in the present study, more sensitive methods, including next-generation sequencing (NGS)-based approaches, have recently emerged. While increased analytical sensitivity may improve detection of very low levels of recipient chimerism, whether this translates into improved prediction of relapse remains to be established. Our findings suggest that the timing of lineage-specific T-cell chimerism assessment is an important determinant of its clinical utility for identifying patients at risk of late relapse, irrespectively of the analytical method employed. Future prospective studies should evaluate whether NGS-based chimerism analysis provides incremental prognostic value beyond conventional STR-based methods, particularly when integrated with MRD assessment.

### Taken together, these findings support a dual-risk model of relapse after HCT

From a clinical perspective, early MTC may identify patients in remission who remain at increased risk of late relapse. This may support risk-adapted strategies, such as intensified monitoring or earlier immunomodulatory interventions, including tapering of immunosuppression or donor lymphocyte infusion. The interval between detection of MTC and overt relapse may represent a clinically relevant window of opportunity for intervention, although the efficacy and safety of such approaches require evaluation in prospective studies.

Interestingly, in vivo T-cell depletion (TCD) was more common in the CTC group, which differs from some previous reports^15,37^. One possible explanation is the higher proportion of HLA-disparity among patients receiving TCD which may enhance early proliferation of alloreactive T cells and facilitate CTC^15,26^. The exposure to other immunosuppressants (e.g. calcineurin inhibitors) and composition of the conditioning regimens (e.g. busulfan-based conditioning) may also influence T-cell chimerism dynamics^26^. These findings highlight the complexity of factors influencing immune reconstitution and chimerism patterns after transplantation.

This study has several limitations. The retrospective design introduces potential residual confounding and selection bias. For a substantial proportion of patients, MRD data were incomplete, limiting statistical power for analyses involving pre-HCT MRD and potentially contributing to the lack of statistical significance in some MRD-related findings. T-cell chimerism was assessed at a single time point and longitudinal kinetics were not available, limiting conclusions regarding the relative importance of persistent or transient MTC. However, an important strength is that chimerism assessment coincided with routine evaluation of remission status and MRD at three months, reducing the likelihood that chimerism status reflected residual disease. In addition, this time point precedes most post-HCT immunomodulatory interventions, such as donor lymphocyte infusion or tapering of immunosuppression, which may otherwise influence both immune reconstitution and relapse. Furthermore, we did not capture detailed longitudinal data on immunosuppression or pre-emptive interventions.

Another limitation is that T-cell chimerism reflects the relative proportion of donor and recipient T cells rather than the absolute number of donor-derived immune effector cells. Nevertheless, by capturing the balance between donor and recipient immunity, it remains a clinically meaningful surrogate marker of immune reconstitution and has been associated with clinical outcomes. Finally, although recent consensus recommendations define mixed donor chimerism as 5–95% donor cells in one or both myeloid and lymphoid lineages^38,39^, our predefined threshold of < 95% donor T-cell chimerism for MTC, is closely aligned with these recommendationas and several previous studies^8,15^, supporting comparability within the excisting AML/MDS literature.

In conclusion, in this large AML cohort, early T-cell chimerism provides prognostic information distinct from pre-HCT MRD and is specifically associated with late relapse after allogeneic transplantation. These findings highlight the potential role of early immune reconstitution in long-term disease control and may help inform future studies aimed at refining post-HCT monitoring and management strategies.

## Supplementary Information

Supplementary Information.

## Data Availability

The dataset analysed during the current study is available from the corresponding author on reasonable request.
