# Supplementary material for: Mixed T-cell chimerism at 3 months predicts late relapse in acute myeloid leukemia after allogeneic stem cell transplantation
Source: Sci Rep. 2026 Sep 4;16:27777. doi: 10.1038/s41598-026-69047-8 (PMC13545257; doi:10.1038/s41598-026-69047-8)
Supplement: Supplementary file 1 [file 41598_2026_69047_MOESM1_ESM.pdf]

SUPPLEMENTARY INFORMATION

Supplementary Methods

Methods for analyzing T-cell chimerism

Based on pre-transplant profiling, the chimerism analyses and assessments were done at the transplant centers´ respective laboratories and according to local and accredited standard operation procedures. Lineage-specific T-cell isolation was performed using immunomagnetic separation (Dynabeads Thermo Fisher Scientific, Waltham, MA, USA). Donor chimerism was analyzed using short tandem repeat (STR) PCR, and in selected cases by quantitative PCR (qPCR). Patients without T-cell chimerism analysis within 70 days - 150 days post-transplant were regarded as missing T-cell chimerism at three months, and therefore excluded. Chimerism was reported as percentage of donor DNA.

Lund
Assessment of donor chimerism by STR. CD3+ lymphocytes were selected by magnetic beads (Dynabeads). The analytical sensitivity was approximately 1%.

Oslo
Assessment of donor chimerism by STR. $CD2^+$ lymphocytes were selected by magnetic beads (Dynabeads CD2). The analytical sensitivity was approximately 1%.

Stockholm
Both qPCR and STR were used to assess donor chimerism, depending on the proportion of recipient derived cells (when >30%, STR was used). CD3+ lymphocytes were selected by magnetic beads (Dynabeads), initially manually, but from April 2013 a robot was introduced. The analytical sensitivity varied between 0.01% to 5% depending on the method.

Umeå
Both qPCR and STR were used to assess donor chimerism, depending on the proportion of recipient derived cells (when >30%, STR was used). CD3+ lymphocytes were selected by magnetic beads (Dynabeads).The analytical sensitivity varied between 0.05% to 0.5% depending on the method.

Uppsala
Assessment of donor chimerism by STR. CD3+ lymphocytes were selected by magnetic beads (Dynabeads). The analytical sensitivity was approximately 1%.

Göteborg
Assessment of donor chimerism by STR. CD3+ lymphocytes were selected by magnetic beads (Dynabeads™). The analytical sensitivity was approximately 1%.
In some cases with sex-mismatch between recipient and donor, FISH was used, with a detection level of 0.2%.

Risk categorization

AML risk categorization according to the Swedish national AML guidelines and ELN2017

Genetic low risk (LR)

- APL with t(15;17)(q24;q21), or molecularly detected PML/RARA-fusion. In rare cases there are variant translocations of RARA present. These are categorized as low risk, also in the presence of other concomitant chromosomal abnormalities.
- inv16(p13q22)/t(16;16)(p13;q22), or molecularly detected CBFB/MYH11–fusion or CBFB-rearrangement. These are classified as low risk, also in the presence of other concomitant chromosomal abnormalities.
- Exception: Not categorized as low risk if KIT-mutation is detected.
- t(8;21)(q22;q22), alternatively molecularly detected RUNX1/RUNX1T1-fusion (formerly AML/ETO). These are categorized as low risk, also in the presence of other concomitant chromosomal abnormalities.
- *Mutated NPM1 concomitant wild type FLT3-ITD and normal karyotype.*
- Double mutation of CEBPA and normal karyotype.

Genetic intermediate risk (IR)

- Normal karyotype without any concomitant abnormalities: FLT3-ITD, mutated NPM1 or double mutation of CEBPA.
- *Normal karyotype with concomitant mutation of both NPM1 and FLT3-ITD.*
- t(9;11)(p21;q23).
- Cytogenetic abnormality (karyotype) neither categorized as low or high risk.

Genetic high risk (HR)

- *Normal or not-riskcategorizing karyotype with concomitant mutation of FLT3-ITD and wild type NPM1.*
- inv(3)(q21q26) or t(3;3)(q21;q26); GATA2::MECOM (formerly EVI1).
- t(6;9)(p22;q34); DEK/NUP214.
- t(v;11)(v;q23); KMT2A-rearrangement (formerly classified MLL-rearrangement). Exception: t(9;11)(p21;q23) is categorized as intermediate risk.
- Isolated del(5q) or -5 or with other concomitant abnormalities.
- Isolated del(7q) or -7 or with other concomitant abnormalities.
- del(17p).
- Complex karyotype, defined as ≥3 chromosomal abnormalities in the absence of t(15;17)(q22;q21), t(8;21)(q22;q22), inv(16)(p13q22)/t(16;16)(p13;q22), or t(9;11)(p21;q23).
- TP53 mutation (usually associated with complex and/or monosomal karyotype)

Special cases: Normal Karyotype (NK), or Not Done (ND), and different findings of NPM1 & FLT3

Cg NK or ND	FLT3	NPM1	Risk group
Cg NK/ND	pos	ND	IR
Cg NK/ND	pos	neg	HR
Cg NK/ND	pos	pos	IR
Cg NK/ND	neg	ND	IR
Cg NK/ND	neg	neg	IR
Cg NK	neg	pos	LR
Cg ND	neg	pos	IR
Cg NK/ND	ND	ND	IR
Cg NK/ND	ND	neg	IR
Cg NK/ND	ND	pos	IR

- FLT3- and NPM1-status are only informative, considering risk categorization, if both are concomitantly analyzed.
- Cytogenetics ND is always categorized as intermediate risk unless mutated FLT3.

Final pre-transplant risk categorization (in the CRF) also includes clinical assessment. Secondary AML (secondary to other hematological disease), therapy related AML, refractory AML (>15% blasts in bone marrow after first cycle of chemotherapy or >2 cycles of chemotherapy needed to achieve complete remission) and all other disease status than CR1 (incl CR2), are always categorized as high risk.

Graft-versus-Host Disease prophylaxis

Prophylaxis of GvHD involved ATG; Thymoglobuline® or ATG-Fresenius®/Grafalon®, mainly for patients with URD, with dosing aimed at 4 - 6 mg/kg and 30 - 40 mg/kg, respectively. Additionally, most patients received aGvHD prophylaxis with a calcineurin inhibitor (cyclosporine or tacrolimus) or sirolimus, in some cases combined with mycophenolate (data not shown). Target levels and timing for tapering of calcineurin inhibitors or sirolimus were at the discretion of the treating physician, based on individual patient and disease characteristics and local SOPs. Besides, GvHD prophylaxis with 2-4 doses of methotrexate (administrated day +1 to +11 after HCT; 16–45 mg/m2), was used.

Conditioning intensity

Conditioning intensity, RIC or MAC, was defined according to Bacigalupo *et al* [30]. Total Body Irradiation ≥8 Gy fractionated and Busulfan >8 mg/kg orally (Bu) or >6,4 mg/kg intravenous (BuS) was regarded as MAC. Treosulfan-based conditioning was defined as RIC (non-myeloablative), when the total treosulfan dose was 30 g/m2, and as MAC (toxicity-reduced) when the total dose was 42 g/m2. No patient received 36 mg/m2 of treosulfan.

Statistical analyses

Chi-square (or Fisher´s Exact) test for categorical variables, Mann-Whitney U-test for ordinal data or if skewed distribution, or the Student's t-test for continuous and normally distributed variables, was used when comparing baseline characteristics between the CTC and MTC, groups.

The median follow-up time was estimated by the reverse Kaplan-Meier method [32,33].

Time-to-event outcomes were analyzed using survival methods.

Landmark Design

All time-to-event analyses were conducted within a pre-specified landmark framework. The landmark time was defined as 3 (L3) or 12 (L12) months after transplantation. Only patients alive and event-free at landmark time were included in the landmark cohort. Follow-up time was measured from the landmark time and patients were followed for a maximum of 60 months thereafter. This approach was chosen to avoid immortal time bias and to ensure that predictors measured post-transplant were assessed in a temporally appropriate manner.

Competing Risks Analyses

Relapse was analyzed using Fine-Gray subdistribution hazard models to account for competing NRM. Time was measured from the landmark time to relapse, NRM, or censoring at last follow-up. Subdistribution hazard ratios (SHR) with 95% confidence intervals (CI) were reported. Cumulative incidence functions were estimated to describe relapse in the presence of competing risks. Death was labelled as competing event in the CIR analysis. In the NRM analysis, relapse was the competing event. Cumulative incidence function curves were generated using Stata software (see below), and group differences were evaluated using Gray's test [34,35].

Relapse-free survival and overall survival

Relapse-free survival and OS were analyzed using Cox proportional hazards regression models within the same landmark framework. Relapse-free survival was defined as time from the landmark to relapse or death from any cause. Overall survival was defined as time from the landmark to death from any cause. Patients were censored at last follow-up or at 60 months after the landmark, whichever occurred first. Kaplan-Meier curves were used to estimate RFS and OS. Group comparisons were performed using the log-rank test. Hazard ratios (HR) with 95% confidence intervals were reported from Cox models.

Model building

Model building followed a pre-specified strategy. Each predictor was first evaluated in univariable models. Multivariable models were then constructed to assess independence of effects and potential confounding. Confounding was evaluated using a change-in-estimate approach within the same analysis population. A change in HR or SHR greater than 10% after adjustment was considered indicative of meaningful confounding. Interaction (effect modification) was assessed by including multiplicative interaction terms and evaluated using a likelihood-ratio test comparing regression models with and without the interaction term. Proportional hazards assumptions were assessed using Schoenfeld residuals.

Two-sided p-values less than 0.05 were considered statistically significant. For comparison of background characteristics, the SPSS version 30 (IBM® SPSS® Statistics, NY, USA) was used. The survival and cumulative incidence analyses were performed using Stata (StataCorp, College Station, TX) version 19.5. Figure 1b was generated using SankeyMATIC (https://sankeymatic.com).

Supplementary Results

T-cell chimerism in peripheral blood (PB) and/or bone marrow (BM)

There was a discrepancy in T-cell chimerism status (MTC or CTC) between BM and PB in 30 of 195 (15%) patients, who had both analyses done simultaneously. Amongst 158 patients with CTC in PB, 24 (15%) had MTC in BM. In reverse, amongst 140 patients with CTC in BM, 6 (4%) patients had MTC in PB.

Time to relapse and from relapse to death

Patients with pre-transplant MRDpos had numerically shorter median time to relapse, and from relapse to death, compared to patients with MTC. This was also seen in patients with both pre-transplant MRDpos and CTC versus pre-transplant MRDneg and MTC. The differences were not significant. See Supplementary Figure 3.

Donor lymphocyte infusions

Among all patients receiving DLI (n=57), 21% (n=12) were administrated due to descending donor T-cell chimerism, of which 83% (n=10) had MTC at 3 months post HCT. Out of these 12 patients, 33% relapsed after DLI, in median 1238 (range:632-2599) days from DLI, and one patient without relapse died due to GvHD-related complications. None of these DLI's were administrated before 3 months post-transplant. Other causes of DLI were relapse, MRD-positivity or clonal evolution (67%), and 9% were not specified.

Conversion in MRD and T-cell chimerism status

No significant difference was seen between patients achieving CTC or MTC regarding conversion from positive MRD at transplant to negative MRD three months post-transplant.

In the CTC group 26/33 (79 %) converted from MRDpos pre-transplant to MRDneg at three months post-transplant compared to 8/12 (67%) in the MTC group (p=0.19). Conversion from MRDneg pre-transplant to MRDpos at three months post-transplant was seen in 3 and 5 patients in the MTC and CTC group, respectively (p=0.48).

Effects of background characteristics on T-cell chimerism

In univariable logistic regression analysis, age at transplant, RIC, HLA match and T-cell depletion were associated with MTC (see Supplementary Table 2). In multivariable analysis (including age at transplant, conditioning intensity, HLA match, stem cell source, CMV status and T-cell depletion), only conditioning intensity and T-cell depletion remained significant (data not shown).

Supplementary Tables

Reduced Intensity Conditioning

Flu150-180 + Bu8/BuS 6.4 (n=191)
Flu90 + TBI2 (n=3)
Flamsa-RIC (n=3)
Flu 125 Cy120 (n=1)
Bu8 Cy120 (n=1)

Myeloablative Conditioning

Cy120 + TBI 10-12 Gy (n=9)
Cy100-120 + Bu16/BuS 9.6-12.8 (n=116)
Flu150-180 + Bu16/BuS 9.6-12.8 (n=37)
Flu 150 + Treo 42 (n=26)
Bu +Holoxan (n=1)

Supplementary Table 1. Conditioning regimens. Bu – busulfan (orally); BuS – busulfan (intravenously); Cy – cyclophosphamide; Flu – fludarabine.

Characteristic	Categories	Odds ratio (95% CI) for MTC	p-value
Sex	Female vs Male	1.18 (0.75 – 1.86)	0.47
Age at transplant	Years	0.98 (0.97 – 1.00)	0.040
Sorror score	0-2 vs ≥3	1.21 (0.71 – 2.08)	0.48
Risk at transplant	LR/IR vs HR	0.93 (0.56 – 1.54)	0.77
Cytogen/mutation risk	LR/IR vs HR	1.26 (0.75 – 2.11)	0.38
Conditioning intensity	RIC vs MAC	2.13 (1.33 – 3.42)	0.002
Stem cell source	PB vs BM	0.51 (0.23 – 1.12)	0.095
Donor type	RD vs URD	0.97 (0.55 – 1.71)	0.92
HLA-match	10/10 vs <10/10	2.28 (0.99 – 5.25)	0.053
HLA-match	8/8 vs <8/8	3.95 (1.18 – 13.21)	0.026
Sex mismatch (pat/don)	Male/Female vs others	0.63 (0.28 – 1.40)	0.25
CMV IgG pos patient	Yes vs No	0.65 (0.39 – 1.08)	0.099
Donor age	Years	0.99 (0.97 – 1.01)	0.38
T-cell depletion	Yes vs No	0.49 (0.30 – 0.79)	0.004
MRD pre-HCT	Neg vs Pos	0.87 (0.47 – 1.80)	0.70
MRD 3 mos post-HCT	Pos vs Neg	1.71 (0.66 – 4.41)	0.27

Supplementary Table 2. Association between background characteristics and MTC at 3 months post-HCT.
Logistic regression. BM – bone marrow; CMV – cytomegalovirus; HR – high risk; IR – intermediate risk; LR – low risk; MAC – myeloablative conditioning; MRD – measurable residual disease; MTC – Mixed T-cell chimerism; Neg – negative; PB – peripheral blood; Pos – positive; post-HCT – post-transplant; pre-HCT – pre-transplant; RIC – reduced intensity conditioning.

Supplementary Figures

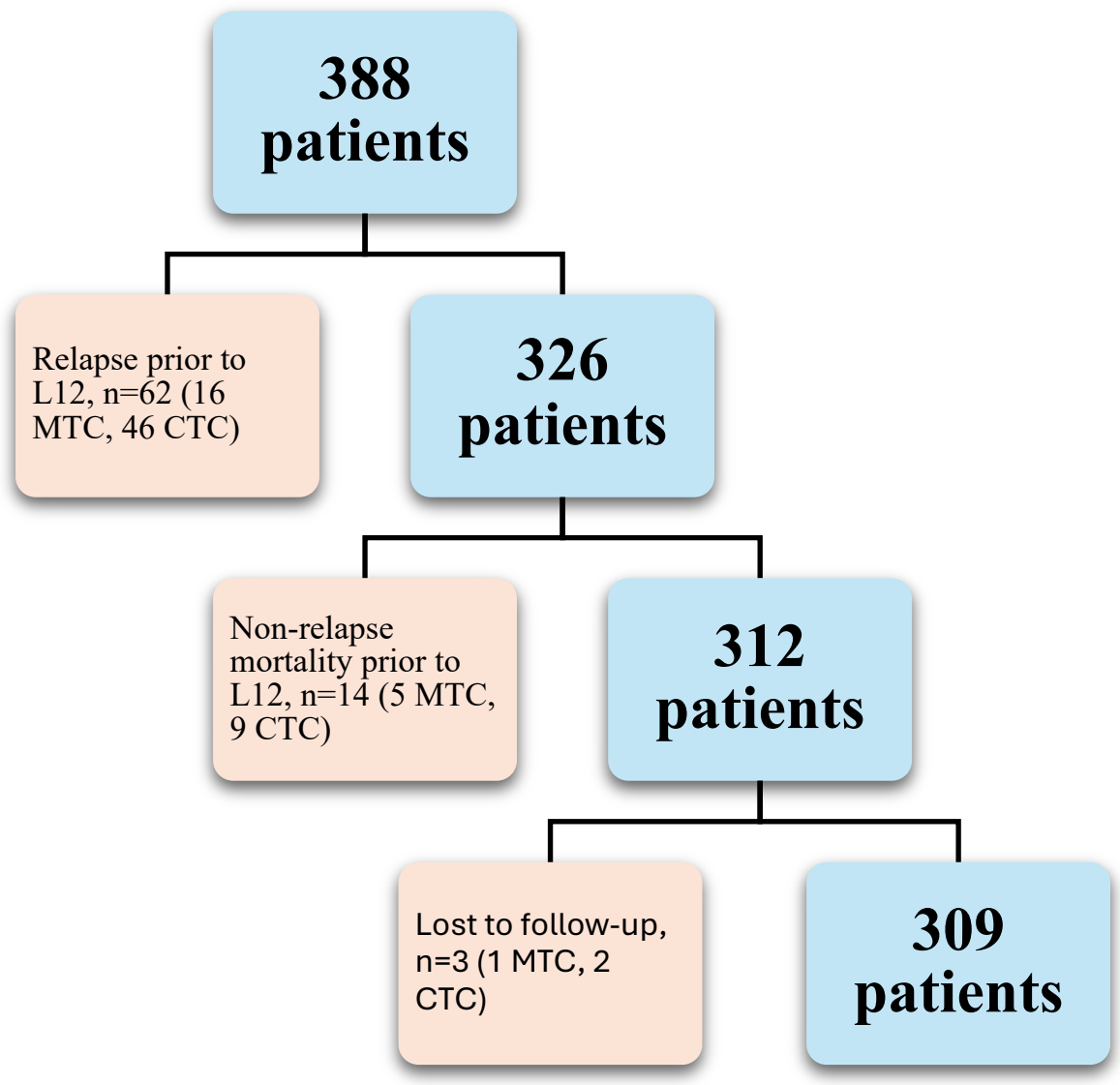

Supplementary Figure 1. Study cohort and landmark populations.
Flow diagram illustrating patient inclusion, exclusions, and populations included in landmark analyses at 12 months (L12) post-HCT. Patients included were in CR at 12 months post-HCT. HCT, allogeneic stem cell transplantation; MTC, Mixed T-cell chimerism at 3 months post-transplant; CTC, Complete T-cell chimerism at 3 months post-transplant; L3, landmark at 3 months post-transplant; L12, landmark at 12 months post-transplant; Lost to F-U, Lost to follow-up; NRM, Non-relapse mortality.

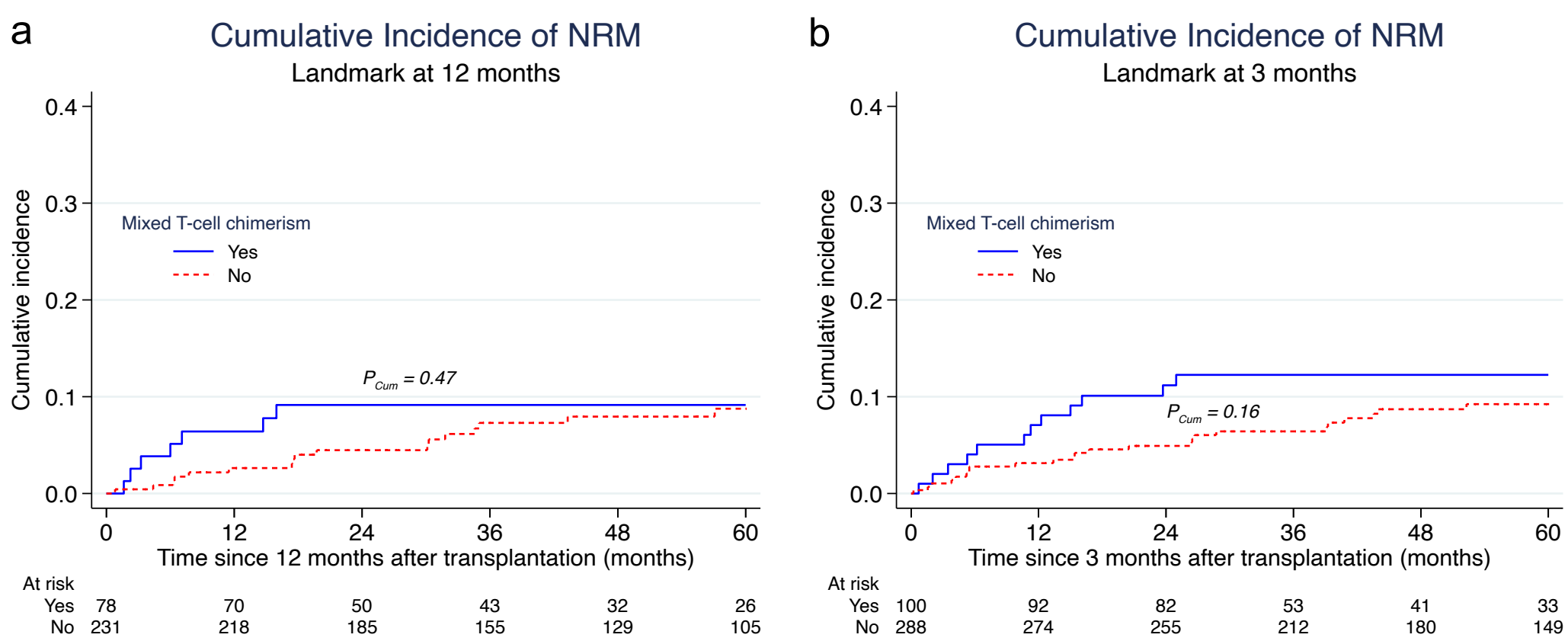

Supplementary Figure 2a and b. The cumulative incidence of non-relapse mortality (NRM) over 60 months after landmark at 12 (A) and 3 (B) months post-transplant, stratified by T-cell chimerism status. Competing event is relapse.

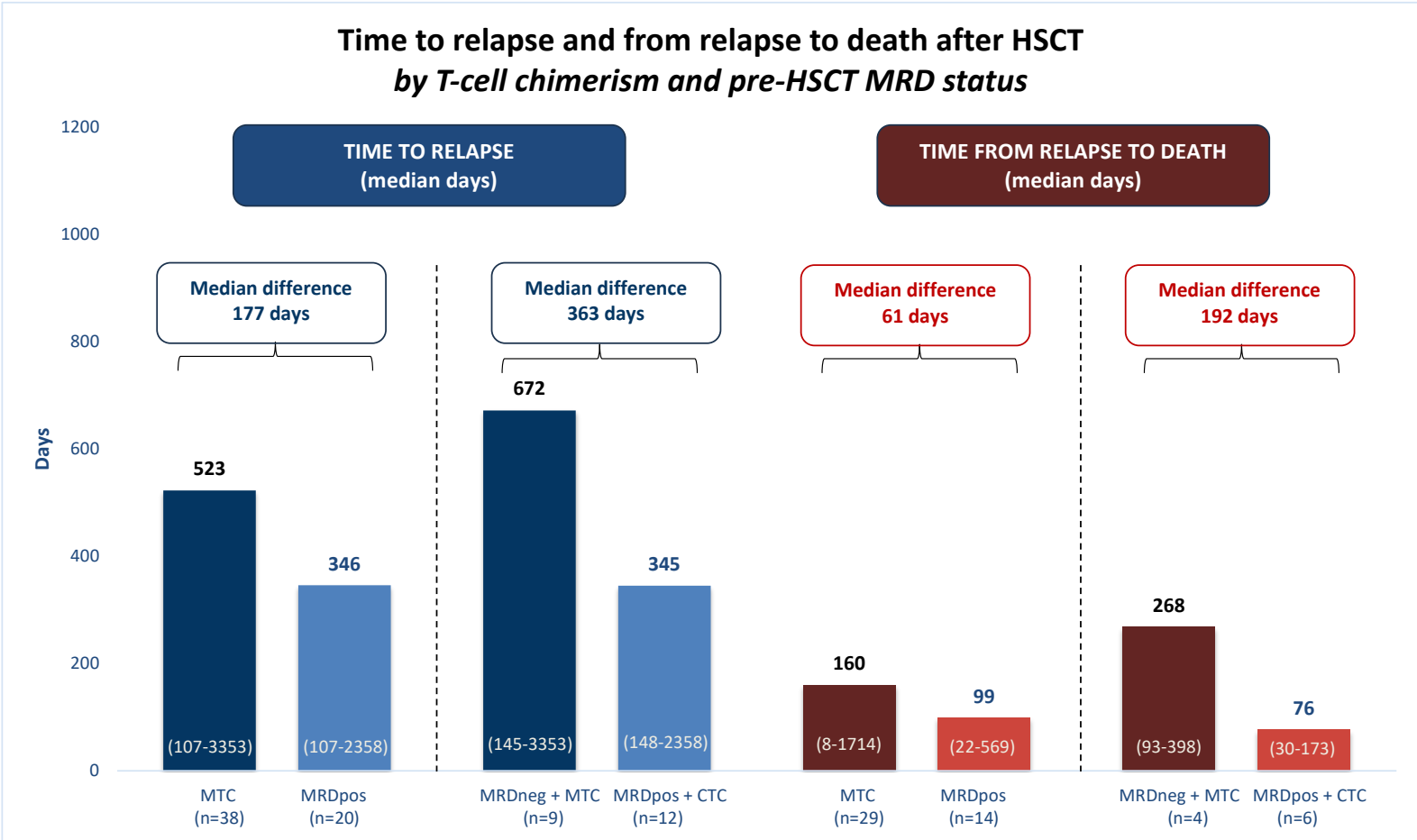

Supplementary Figure 3. Time (median days and range in boxes) to relapse (blue) and b) from relapse to death (red), compared between patients with MTC versus MRDpos and patients with MRDneg plus MTC versus MRDpos plus CTC, respectively. CTC – complete T-cell chimerism; HCT – allogeneic stem cell transplantation; MTC – mixed T-cell chimerism; MRD – measurable residual disease; MRDneg – negative MRD pre-transplant; MRDpos– positive MRD pre-transplant.
